# Molecular Mechanisms of Exopolysaccharide from *Aphanothece halaphytica* (EPSAH) Induced Apoptosis in HeLa Cells

**DOI:** 10.1371/journal.pone.0087223

**Published:** 2014-01-23

**Authors:** Yu Ou, Shuya Xu, Dandan Zhu, Xuegan Yang

**Affiliations:** School of Life Science and Technology, China Pharmaceutical University, Nanjing, China; Indiana University School of Medicine, United States of America

## Abstract

The present study aims to investigate the pharmacological effect of the exopolysaccharides from *Aphanothece halophytica* GR02 (EPSAH) on the HeLa human cervical cancer cell line. HeLa cells were cultured in RPMI-1640-10% FBS medium containing with or without different concentrations of EPSAH. Cell viability was assessed by methylthiazol tetrazolium (MTT) assay. Cell apoptosis was elevated with Wright-Giemsa staining, AO/EB double staining, and DNA fragmentation assay. Apoptosis-associated molecules from cultured HeLa cells were quantified using Western blot analysis. Our results suggest that EPASH induces apoptosis in HeLa cells by targeting a master unfolded protein response (UPR) regulator Grp78. Grp78 further promotes the expression of CHOP and downregulates expression of survivin, which leads to activate mitochondria-mediated downstream molecules and p53-survivin pathway, resulting in caspase-3 activation and causing apoptosis. These findings provide important clues for further evaluating the potential potency of EPSAH for use in cancer therapy.

## Introduction

Exopolysaccharides are high-molecular-weight sugar polymers secreted by microorganisms into the surrounding environment. *Aphanothece halophytica* is a halotolerant cyanobacterium that can grow in a wide range of salinity conditions (0.25 to 3.0 M NaCl), as well as at alkaline pH [1]. The *Aphanothece halophytica* GR02 is known to produce large amounts of exopolysaccharide that exhibits xanthan-like physical properties and is of industrial uses [2]. In addition, studies have demonstrated that the exopolysaccharide from *Aphanothece halophytica* GR02 (EPSAH) possesses potent antitumor, immunomodulatory and antiviral activities [3].

Although it has been reported that oral administration of EPSAH significantly inhibited Sarcoma 180 growth in mice, little is known about the mechanism of its antitumor activity. After an anticancer screening test *in vitro*, we found that EPSAH inhibits the growth of various cancer cells (data not shown). Although these studies revealed that EPSAH acted as an anti-cancer agent, its mechanism of action remains unknown.

Aberrant regulation of apoptosis has been observed in major human diseases, including cancer. Many therapeutic agents inhibit tumor cells by inducing apoptotic cell death. Mitochondria play an important role in cell apoptosis [4]. Apoptotic stimuli induce the opening of the membrane permeability transition (MPT) pore, resulting in the loss of the mitochondrial membrane potential, swelling of the mitochondrial matrix, and breakdown of the mitochondrial membrane and eventually inducing mitochondrial-mediated apoptosis [5], [6]. On the other hand, endoplasmic reticulum (ER) stress activates a complicated signal transduction pathway known as the unfolded protein response [7]. A number of studies have reported UPR activation in a variety of solid tumors and tumor cell lines [8]. The present study examined the effects of EPSAH on growth inhibition and apoptotic induction in HeLa cells. We further investigated the molecular mechanism of EPSAH-induced apoptosis in HeLa cells by determining the expression of the key components involved in the mitochondria-mediated apoptosis and UPR pathways.

## Materials and Methods

### Reagents

RPMI-1640 medium (Gibco, USA), fetal bovine serum(FBS, Gibco, USA), MTT (3-(4,5-dimethylthiazol-2-yl)-2,5-diphenyl tetrazolium bromide, Fluka, USA), acridine orange (AO, Amresco, USA), ethidium bromide (EB, Sigma, USA), Wright-Giemsa dye solution (Nanjing Jiangcheng Bioengineering Institute, Nanjing, China), RNase A (Fermantas, Canada), proteinase K (Fermantas, Canada), ECL plus kit (Beyotime Institute of Biotechnology, China), DL 2000 DNA Marker (Takata, Japan), prestained protein marker (fermentas, Thermo Scientific, USA), antibody to GAPDH, GRP78, GADD153, Bcl-2, Bax, p53, survivin, Actived-Caspase 3 p17 (Bioworld Technology, MN, USA), horseradish peroxidase (HRP)-conjugated secondary antibody (MultiSciences, China). All other biochemicals and chemicals used in the experiment were of analytical grade.

### Culture of Aphanothece halophytica GR02

*Aphanothece halophytica* GR02 was kindly provided by Associate Professor Pengfu Li, Nanjing University. The alga cells were cultivated in the 1M NaCl medium [9], modified by supplementation of trace element solution with A5 and B6 [10]. The algal cells were incubated at 30°C under light irradiation of 4000 lux and a 12 h/12 h light/dark cycle in 1 L conical flasks containing 600 ml of medium. The cultures were continuously aerated by gentle bubbling with filtered air.

### Isolation and purification of EPSAH

After 20 days of culture, the cells were removed by centrifugation. The supernatant was filtered through a 0.45 µm porous membrane, dialyzed against tap water (for 60 h) and distilled water (for 18 h), and then concentrated under reduced pressure at 60°C. The concentrated sample was loaded onto an anion-exchange column filled with DEAE-Sepharose FF (GE Healthcare). The sample was eluted by a linear gradient of 0 to 2 M NaCl in 10 mM phosphate buffer at pH 7. The major peak were collected, dialyzed, concentrated, and then precipitated by the addition of four volumes of 95% ethanol at 4°C overnight, and lyophilized. Total polysaccharides content of the fractions was determined by the phenol-sulphuric acid method [11].

### Spectroscopic analysis of EPSAH

UV–VIS absorption spectra of EPSAH aqueous solution were measured on a Perkin-Elmer Lambda 2 UV/VIS spectrometer. Infrared spectra were recorded on KBr discs with a Nicolet 170 SX IR Spectrophotometer.

### HeLa cells culture and treatments

HeLa cell line was purchased from Shanghai Institute of Cell Biology (Shanghai, China). HeLa cells were cultured in RPMI-1640 medium supplemented with 10% FBS, 100 U/ml penicillin and 100 µg/ml streptomycin and incubated at 37°C in a 5% CO_2_ incubator. Cells were split twice a week by trypsinization at 80–90% confluency and were always used within two months after their removal from liquid nitrogen storage. The cells were seeded in a 96-well microplate or 6-well plates in RPMI-1640 medium containing different concentrations of EPSAH, cultured for 24, 48 or 72 hours.

### Measurement of HeLa cell proliferation

Cell viability was assessed by the MTT assay. After the cell was treated as described in the previous section, 10 µl of 5 mg/ml methylthiazol tetrazolium (MTT, 5 mg/ml) was added to each well, and incubation proceeded at 37°C for another 4 h. The formazan granules obtained were then dissolved in 100 µl DMSO, and absorbance at 570 nm was measured with an ELISA plate reader (Multiskan Mk3, Finland). The percentage of cell survival was then calculated for each group by normalization of the readings against the absorbance of untreated control HeLa cells, which was designated as 100% cell survival.

### Wright-Giemsa and AO/EB staining

Monolayer cultures in 96-well plates were used for these studies. After removal of the culture medium, cells were treated with Wright-Giemsa dye solution or AO/EB (100 µg/ml in PBS) in situ. Cells were examined by light or fluorescence microscopy (OlympusIX51, Japan).

### DNA fragmentation assay

Hela cells were suspended in 10 mM Tris–HCl buffer (pH 8.0) containing 1 mM EDTA and lysed by incubation in the same buffer with 20 µg/ml RNase A, 75 mM NaCl, 100 µg/ml proteinase K, and 0.5% Triton X-100. After 3 h incubation at 50°C, the sample was centrifuged at room temperature, and DNA was extracted by phenol purification and ethanol precipitation. Finally, the DNA samples was separated using a 2.0% agarose gel and photographed under ultraviolet illumination.

### Measurement of the mitochondrial transmembrane potential (ΔΨm)

The mitochondrial transmembrane potential was measured using rhodamine 123 (Rh123) staining and flow cytometry analysis. After treatment as described in the previous section, about 10^6^ cells were collected, washed twice with PBS, and incubated with 10 µg/ml Rh123 for 30 min at 37°C in the dark. Fluorescent intensities were determined by flow cytometry with excitation and emission wavelength set at 488 nm and 530 nm, respectively.

### Western blot analysis

Cell pellets were resuspended in a lysis buffer (50 mM Tris-HCl, 150 mM NaCl, 1% NP-40, 0.1% SDS, and 1 mM PMSF), incubated on ice for 30 min with occasional vortexing, and clarified by centrifugation at 10,000x g for 10 min at 4°C. Samples were resuspended in Laemmli buffer, separated electrophoretically by SDS-PAGE (10–12% gels), and subsequently transferred to a 0.45 µm polyvinylidene difluoride (PVDF) membrane. Following transfer, membranes were blocked in TBST buffer (20 mM Tris–HCl, pH 7.5, 150 mM NaCl, and 0.15% Tween 20) containing 5% (w/v) nonfat milk powder for 1 h and then were incubated overnight at 4°C with the appropriate specific primary antibodies diluted in TBST buffer. The membranes were then washed and incubated in the presence of horseradish peroxidase-conjugated secondary antibodies for 60 min at room temperature. Immunoreactive bands were detected by enhanced chemiluminescence using the ECL plus kit.

### Statistical Analysis of the Data

The data was analyzed using ANOVA and an unpaired Student's t-test. Differences were considered statistically significant for *p* values lower than 0.05. All values were expressed as “mean value ± Standard Deviation (S.D.)”.

## Results

### Purification of EPSAH

The elution profile of chromatography of EPSAH on DEAE-Sepharose Fast Flow is shown in Figure S1. Single polysaccharide peak was obtained and no proteins were detected in the sample. The UV spectra did not show any absorption peaks of protein, nucleotides, or pigments in the wavelength range of 600 to 240 nm (Figure S2A). FT-IR spectra of EPSAH showed several distinct peaks at wavenumbers of 3495 (hydroxyls), 2936 (aliphatic chains), 1614 (carbonyls), 1418 (aliphatic chains), and 1025–1082 (hydroxyls) (Figure S2B), consistent with a sugar content.

### EPSAH inhibits cell growth and induce apoptosis in HeLa cells

**Figure 1:**
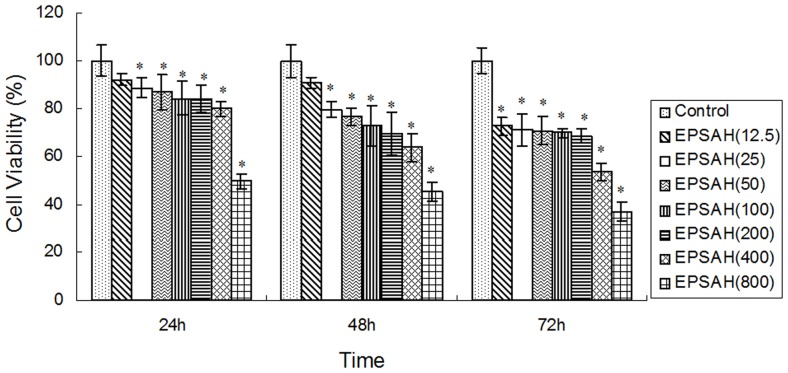
Effect of EPSAH on the cell proliferation in human HeLa cells. HeLa cells were seeded in 96-well culture plates. After incubation for 24, 48 or 72 h, they were subjected to MTT assay. Results are expressed as percent cell proliferation relative to the proliferation of control. Data represents as mean ± S.D. from three independent experiments. *P<0.01 *vs* control.

When HeLa cells were exposed to various concentrations (12.5–800 µg/ml) of EPSAH for 24, 48 or 72 h, EPSAH treatment clearly reduced Hela cell viability in a time- and dose-dependent manner (Fig.1).

**Figure 2:**
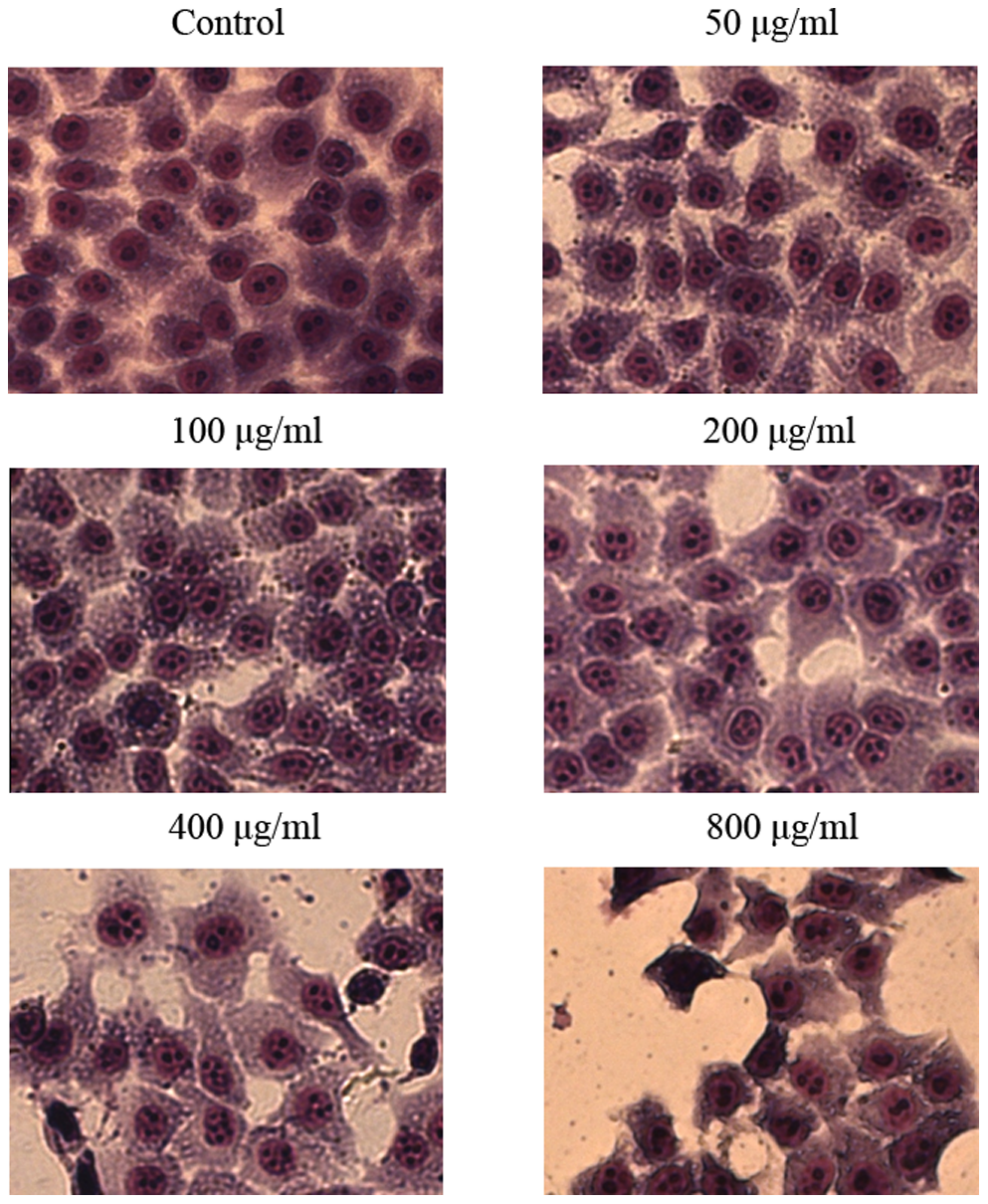
Morphological changes in HeLa cells treated with different concentrations of EPSAH (50–800 µg/ml) for 48 h and compared with untreated cells. The cells were examined under the light microscope after Wright-Giemsa stain. Nuclear fragmentation, chromatin condensation and/or chromatin margination was observed in EPSAH-treated HeLa cells. The most representative fields are shown.

Analysis of Hela cells using Wright-Giemsa stain showed that EPSAH treatment (50–800 µg/ml for 48 h) caused evident nuclear fragmentation, chromatin condensation and/or chromatin margination (Fig. 2), all of which were characteristic morphological alterations associated with apoptosis.

**Figure 3:**
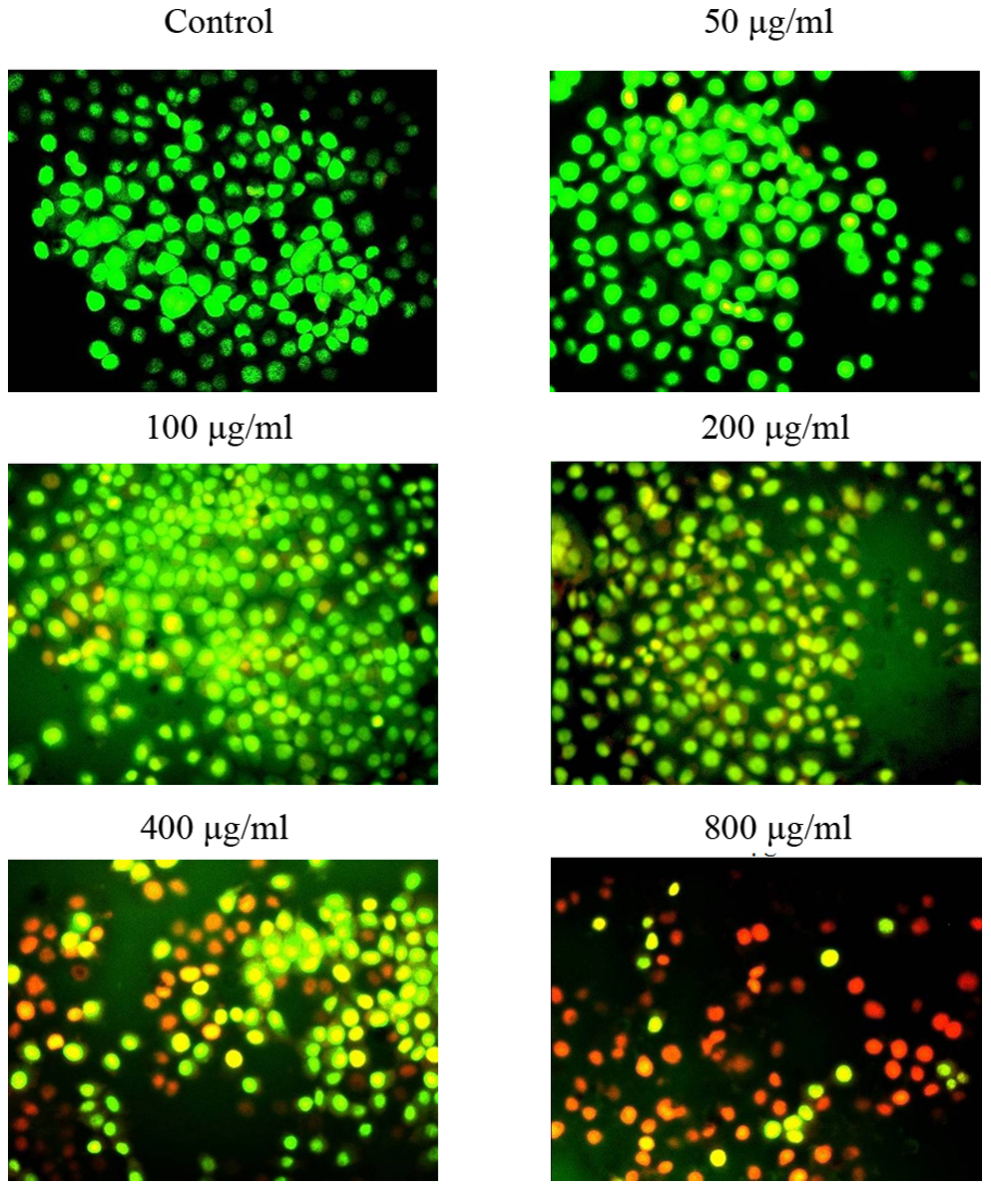
Identification of apoptotic cells by AO/EB staining. HeLa cells were treated with different concentrations of EPSAH (50–800 µg/ml) for 48 h and compared with untreated cells. The most representative fields are shown.

**Figure 4:**
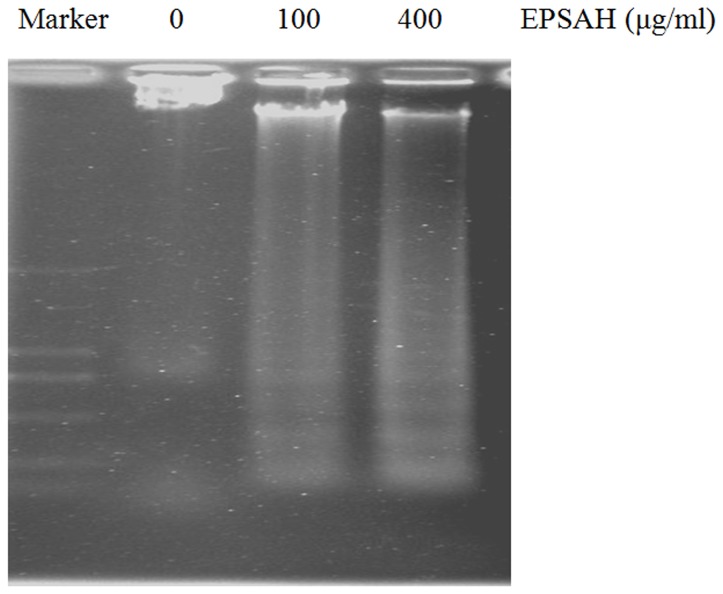
Agarose gel etectrophoresis of DNA from HeLa cells treated for 48 hours with different concentrations of EPSAH.

AO/EB double staining combines differential uptakes of fluorescent DNA binding dyes AO and EB, allowing one to distinguish viable, apoptotic, and necrotic cells: live viable cells (green), live apoptotic cells (orange), and dead (necrotic) cells (red) [12], [13]. Hela cells treated with EPSAH for 48 h showed an increased number of orange- and red-stained cells in a dose-dependent manner (Fig. 3). Moreover, Hela cells exposed to 100 and 400 µg/ml of EPSAH for 48 hours showed significant DNA fragmentation as revealed by apoptotic DNA ladder (Fig. 4).

### EPSAH-induced activation of caspase 3 in HeLa cells

**Figure 5:**
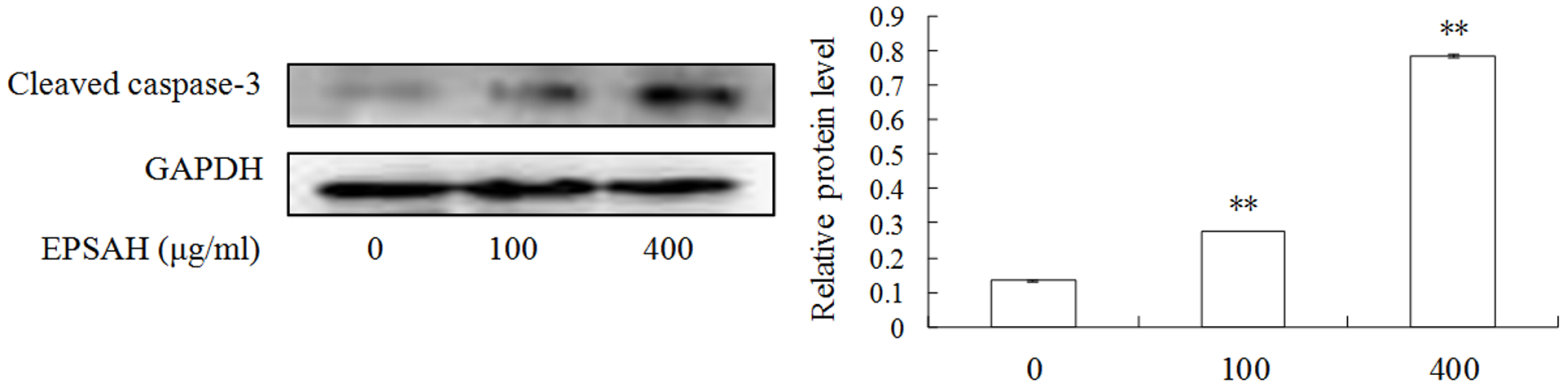
Effect of EPSAH on the level of the cleaved caspase-3 in HeLa cells. The level of the cleaved caspase-3 were assayed by Western blotting in the cells treated with EPSAH at the indicated doses for 48 h.

The caspase family of cysteine proteases plays a key role in apoptosis. Caspase 3 is the most extensively studied apoptotic protein among caspase family members [14]. So we examined the activation of caspase-3 in response to EPSAH treatment. As expected, EPSAH increased the levels of the cleaved caspase-3 in HeLa cells in a dose-dependent manner (Fig. 5).

### EPSAH-induced loss of mitochondrial transmembrane potential (ΔΨm) in HeLa cells

**Figure 6:**
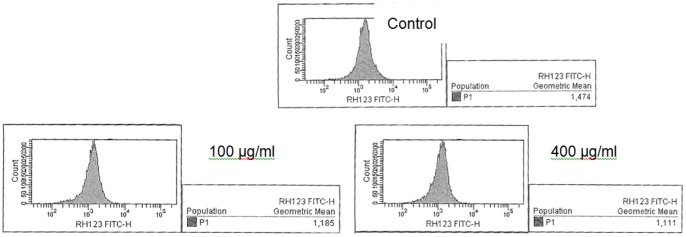
EPSAH-induced mitochondrial transmembrane potential in HeLa cells measured by Rh123 staining and analyzed by flow cytometry.

Rh123 accumulates in normal mitochondria due to its high negative charge, and the reduction of ΔΨm will lead to the release of Rh123 and reduction of its fluorescence intensity. As shown in Figure 6, after staining with Rh123, HeLa cells treated with different concentrations of EPSAH for 48 h exhibited a lower Rh123 staining compared with untreated HeLa cells.

### EPSAH modulated the expression of proteins involved in DNA damage and apoptosis in HeLa cells

**Figure 7:**
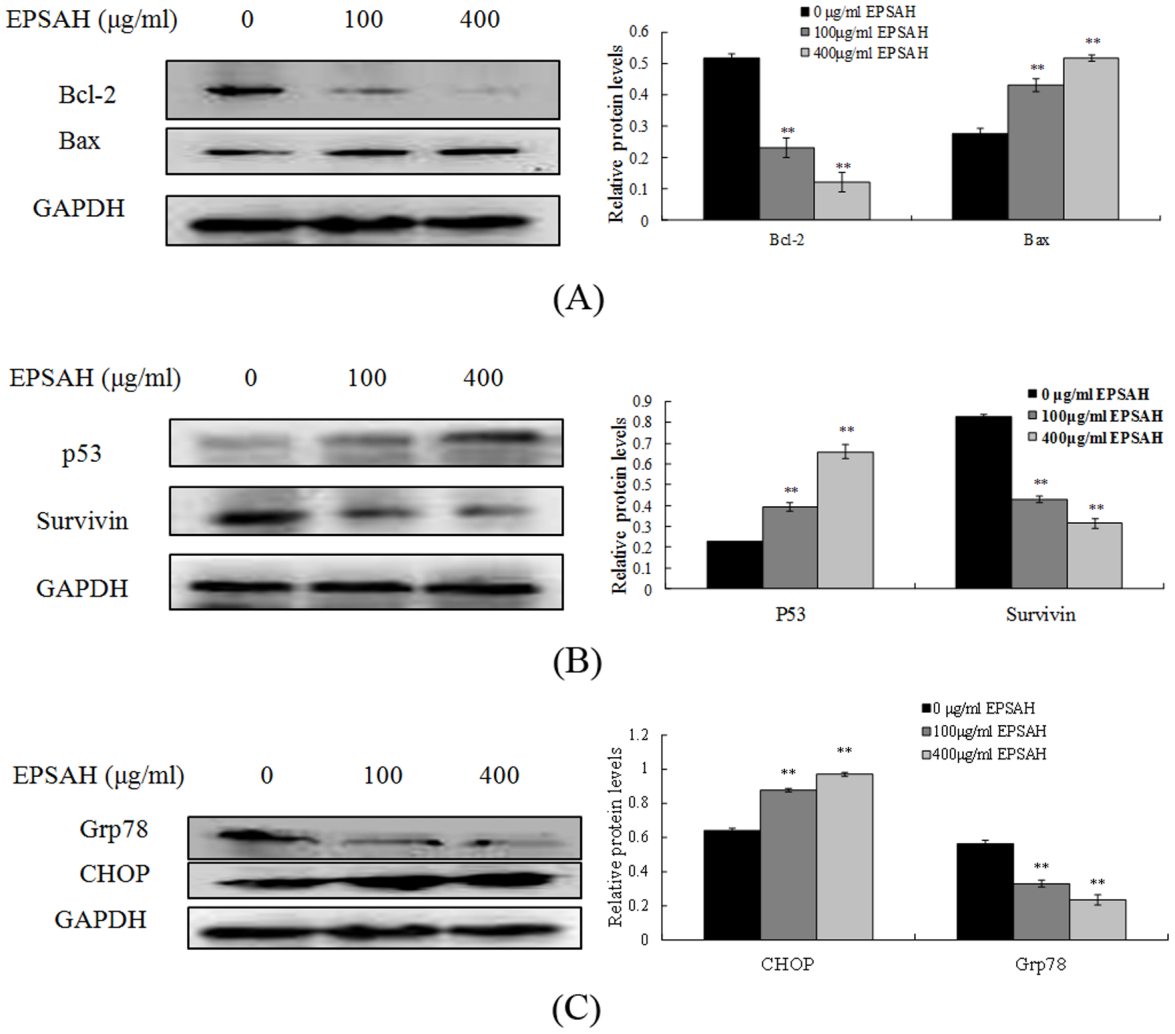
EPSAH induced Bcl-2, Bax, P53, survivin, Grp78 and CHOP protein expression levels in HeLa cells. Cells were treated with EPSAH at the concentrations of 100 and 400 µg/ml for 48 h. The treated and untreated cells were lysed and cell extract subjected to western blots with the antibodies as described in the Methods section. Immunoblot images are the representative of 3 identical experiments. The GAPDH blot is a loading control. (A) Bax/Bcl-2 proteins, (B) P53 and survivin proteins (C) Grp78/CHOP proteins.

Western blot analysis was further utilized to determine the effect of EPSAH on the proteins related to DNA damage and apoptosis in HeLa cells. As shown in Figure 7A, EPSAH treatment led to an increase in Bax, a pro-apoptotic protein and a marked decrease in Bcl-2, an anti-apoptotic protein. Consistent with these findings, the expression level of p53 protein was significantly upregulated while survivin, an inhibitor of apoptosis was down-regulated in cells treated with EPSAH in comparison with untreated control cells (Fig. 7B). In addition, the cellular level of CHOP/GADD153 (growth arrest and DNA damage inducible gene 153), a protein involved in ER stress-induced cell death, was significantly increased whereas the expression of Glucose regulated protein 78 (Grp78), also referred to as immunoglobulin heavy chain binding protein (BiP), was significantly attenuated in the cells exposed to EPSAH (Fig. 7C). GRP78 is an ER molecular chaperone that is ubiquitously expressed and plays an important role in the development and progression of cancer [15].

## Discussion

Our study demonstrates that EPSAH suppresses cell growth and induces apoptosis in HeLa cell, a human cervical cancer cell line. EPSAH-induced apoptosis of HeLa cells was demonstrated by Wright-Giemsa and AO/EB staining, DNA Ladder formation and caspase 3 activation. All results suggest that apoptosis is the major form of HeLa cell death caused by EPSAH exposure, and the effects appear to be dose- and time-dependent. Based on these findings, we further investigated the potential mechanism associated with ESPAH-mediated apoptosis in HeLa cells.

Our study shows that apoptosis induced by EPSAH exposure is associated with an upregulation of Bax and simultaneous downregulation of Bcl-2, eventually leading to an increase in the ratio of Bax/Bcl-2 proteins. The Bcl-2 family proteins Bax and Bcl-2 play important roles in initiating the mitochondria-mediated apoptotic pathway [16]. Pro-apoptotic protein Bax translocates to the mitochondria and integrates into the outer mitochondrial membrane, where it promotes the disruption of ΔΨm and the release of cytochrome c into the cytosol and ultimately the activation of caspase 3 [17]. In contrast, anti-apoptotic protein Bcl-2 prevents this process by preserving mitochondrial integrity. Thus, the ratio of Bax to Bcl-2 is crucial to the mitochondria-mediated apoptotic pathway [18]. Consistent with the increased ratio of Bax/Bcl-2, a marked loss of ΔΨm was also observed in HeLa cells treated with EPSAH. Taken together, these results suggest that mitochondria-mediated apoptotic pathway is involved in EPSAH-induced HeLa cell death.

P53 is activated when DNA damage occurs or cells are stressed and is translocated to the nucleus, where it can induce proapoptotic gene expression, on the mitochondrial membrane and activate the effector caspases and accelerate cell death [19]. P53 directly activates Bax to permeabilize mitochondria and engage the apoptotic program [20]. On the other hand, Survivin is the smallest member of the inhibitor of apoptosis (IAP) gene family, synergistically inhibits caspase-3 and -9, suppresses apoptosis, and accelerates tumor progression [21]. As a downstream factor highly expressed in cancer and regulated by P53, survivin is a dual mediator of resistance to apoptosis and cell cycle progression. Thus, regulation of the p53-survivin signal pathway is important for cell survival [22]. Little is known regarding the effect of EPSAH on this pathway. Our results show that EPSAH promotes the levels of P53 and suppresses the levels of survivin in HeLa cells. This is in agreement with other reports that survivin is a target of and negatively regulated by P53 [23]. These finds further suggest that EPSAH may induce Hela cell apoptosis through modulating the p53-survivin pathway.

Acute stress responses are strongly associated with the UPR, the acute responsive cells that undergoes apoptosis via the UPR target CHOP, which inhibits the expression of the anti-apoptotic gene bcl-2 and activates pro-apoptotic gene expression [24]. This indicates that there is a crosstalk between mitochondria pathway and UPR pathway. Grp78 serves as a master UPR regulator that plays a central role in modulating its downstream signaling and is overexpressed and mis-localized in many types of tumor cells. The level of Grp78 may be used as an effective marker to indicate aggressive behavior and prognosis of prostate cancer and gastric carcinomas [25], [26] and is preferably required for tumor proliferation, survival, and angiogenesis. It has also been shown to suppress CHOP induction and ER stress–induced apoptosis in tumors [27], [28]. In HeLa cells, EPSAH treatments diminish the protein levels of Grp78 while promoting the protein levels of CHOP. These results suggest that a potential crosstalk between mitochondria and UPR pathways EPSAH mediated apoptosis, in which CHOP induction may inhibits the expression of the anti-apoptotic gene bcl-2.

One of the functions of survivin in the cellular stress response is beginning to emerge, and involves the association of survivin with various molecular chaperones. These interactions may promote adaptation under conditions of cellular stress by maintaining survivin protein stability [21]. Here, we find that EPSAH treatment downregulated expression of the Grp78 and survivin, demonstrating the association of survivin with the molecular chaperone Grp78 in HeLa cells.

**Figure 8:**
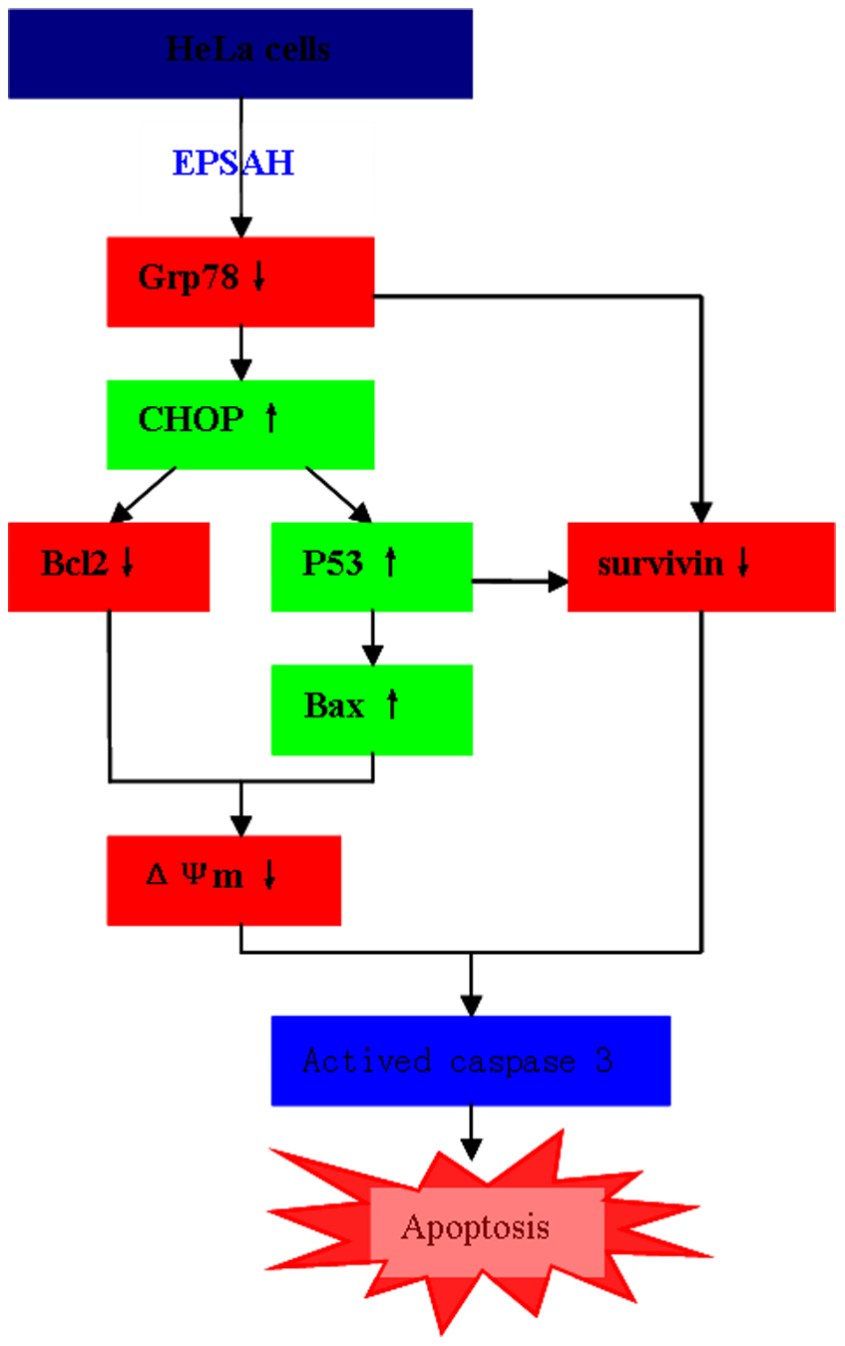
A potential mechanism of EPSAH-induced cell apoptosis in HeLa cells. (↑) and (↓) arrows indicate increase and decrease.

In summary, our results reveal a possible molecular mechanism for EPSAH-induced cell apoptosis in HeLa cells as outlined in Figure 8. In this model, EPSAH first targets a master UPR regulator Grp78, promoting the protein levels of CHOP and downregulating the expression of survivin, which activate mitochondria-mediated downstream molecules and p53-survivin pathway, and eventually leads to the activation caspase-3 and apoptosis. Collectively, these findings provide important clues for further evaluating EPSAH as a potential anti-cancer therapy.

## Supporting Information

Figure S1 **Elution profile of EPSAH.** The chromatogratic separation of EPSAH from A. *halophytica* on a DEAE-SFF colume (3.0 cm×15 cm) eluted with a linar gradient of 0.1 to 2 mol/L NaCl with a flow rate of 3 ml min^−1^.
(TIF)

Figure S2 **EPSAH Spectra.** (A) UV–vis spectrum (B) FT-IR spectrum.
(TIF)
