# Supplementary figures and images for: Molecular Mechanisms of Exopolysaccharide from Aphanothece halaphytica (EPSAH) Induced Apoptosis in HeLa Cells
Source: PLoS One. 2014 Jan 23;9(1):e87223. doi: 10.1371/journal.pone.0087223 (PMC3900761; doi:10.1371/journal.pone.0087223)

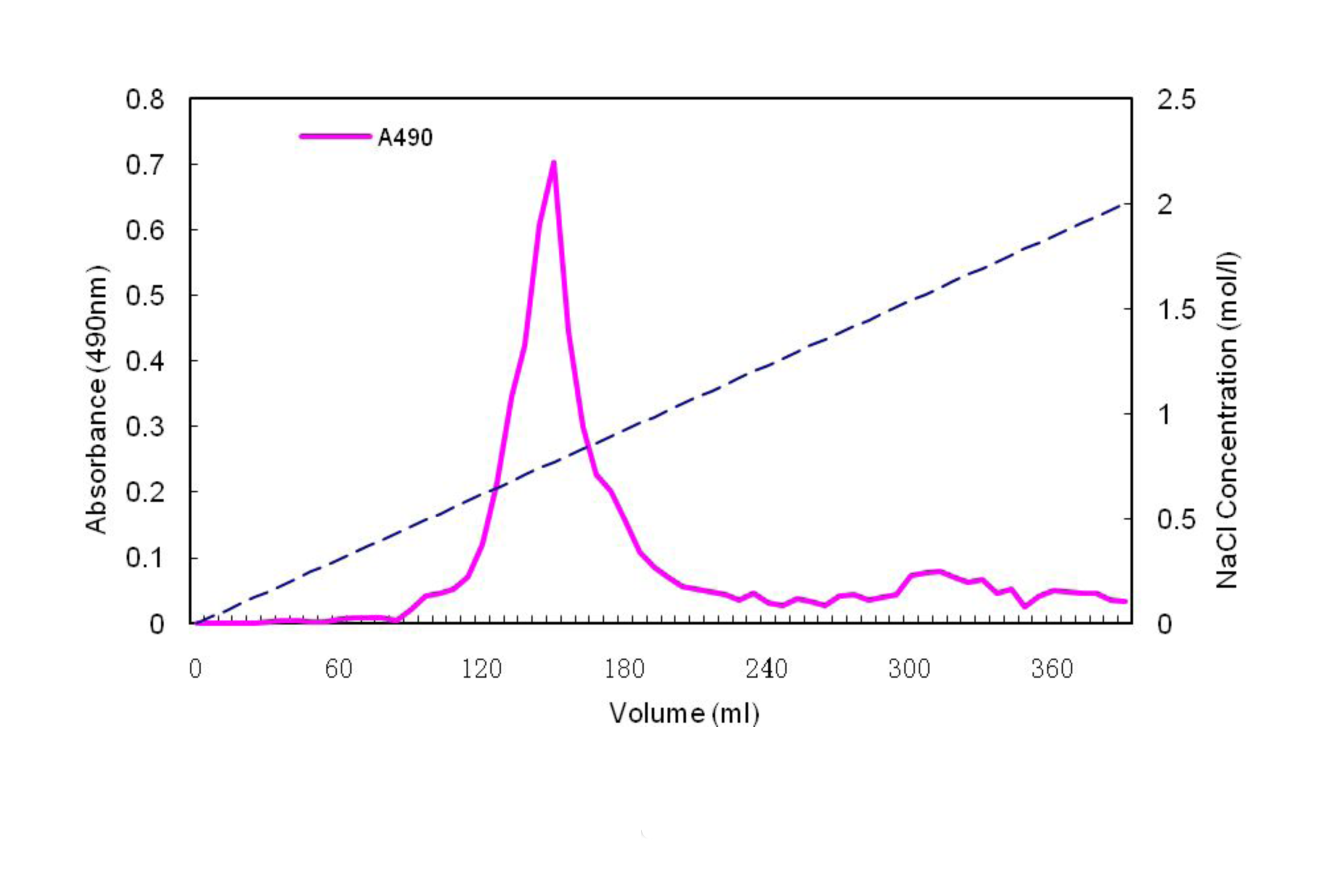

Supplement: Figure S1 — Elution profile of EPSAH. The chromatogratic separation of EPSAH from A. halophytica on a DEAE-SFF colume (3.0 cm×15 cm) eluted with a linar gradient of 0.1 to 2 mol/L NaCl with a flow rate of 3 ml min−1. (TIF) [file pone.0087223.s001.tif]

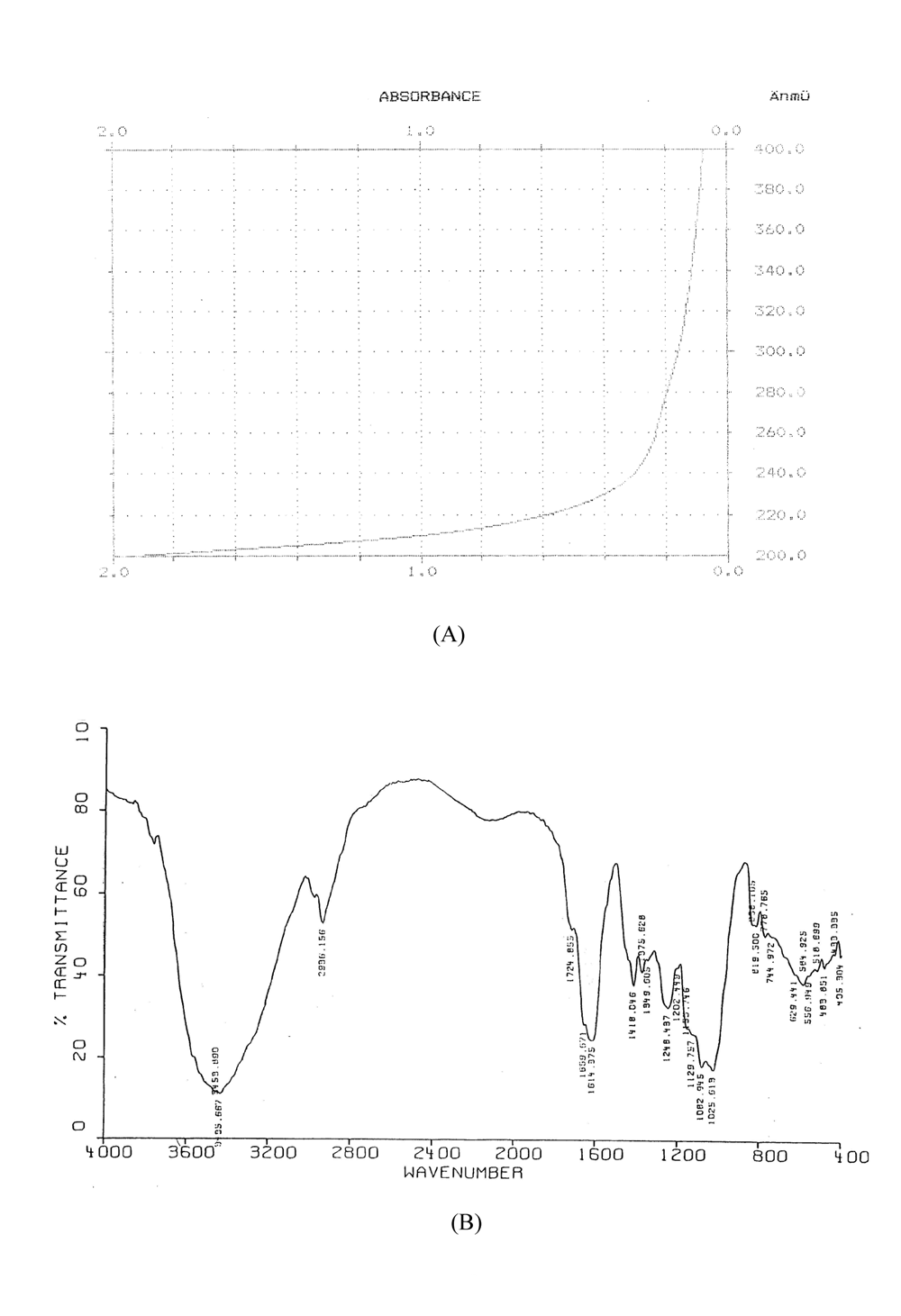

Supplement: Figure S2 — EPSAH Spectra. (A) UV–vis spectrum (B) FT-IR spectrum. (TIF) [file pone.0087223.s002.tif]
